# Supplementary material for: High Expression of miR-34a Associated with Less Aggressive Cancer Biology but Not with Survival in Breast Cancer
Source: Int J Mol Sci. 2020 Apr 26;21(9):3045. doi: 10.3390/ijms21093045 (PMC7246662; doi:10.3390/ijms21093045)
Supplement: Supplementary file 1 [file ijms-21-03045-s001.pdf]

Supplementary Table 1. Clinicopathological demographics of the miR-34a High and miR-34a Low groups in TCGA cohort.

Clinicopathological Factor	Whole Cohort (*n* = 755)		
	miR-34a High n = 377	miR-34a Low n = 378	*p* value
**Age**			
<65 y	250	98	0.0257
≥65 y	127	279	
Unknown	0	1	
**Stage**			
1	68	69	0.432
2	215	215	
3	83	88	
4	7	2	
Unknown	4	4	
**T category**			
1	99	113	0.067
2	220	203	
3	41	55	
4	16	7	
Unknown	1	0	
**Lymph node metastasis**			
Negative	180	177	0.826
Positive	191	196	
Unknown	6	5	
**ER Status**			
Negative	88	77	0.215
Positive	260	284	
Unknown	29	17	
**PgR Status**			
Negative	120	104	0.107
Positive	227	256	
Unknown	30	18	
**HER2 Status**			
Negative	257	278	0.32
Positive	66	58	
Unknown	54	42	
**PAM 50 classification**			
Normal	10	6	< 0.001
Luminal A	101	149	
Luminal B	74	40	
HER2	25	18	
Basal	42	41	
Unknown	125	124	
**Histologic subtype**			0.002
Invasive Ductal	272	246	

Carcinoma		
Invasive Lobular Carcinoma	67	109
Mixed Ductal and Lobular Carcinoma	10	8
Other	22	12
Unknown	6	3

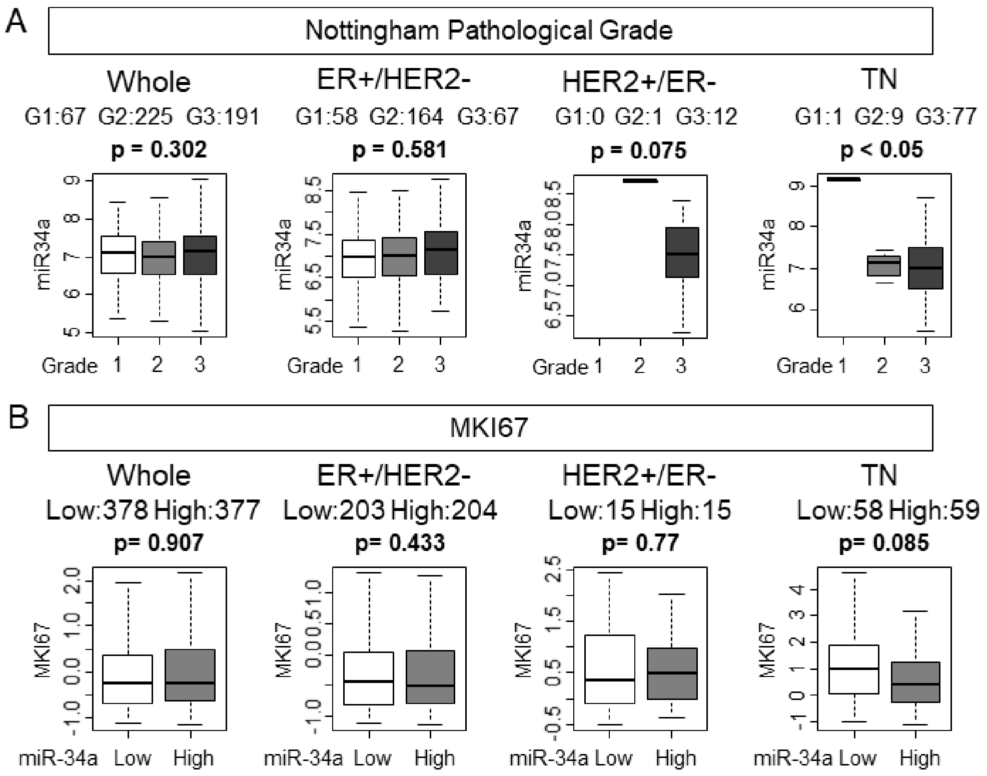

Supplementary Figure S1: The association of miR-34a expression with Nottingham pathological grade and the expression of MKI67 in TCGAC cohort. (A) Association between miR-34a expression levels and Nottingham pathological grade in whole cohort and each subtype. (B) Association between expression levels of miR-34a and MKI67 expression in whole cohort and each subtype.

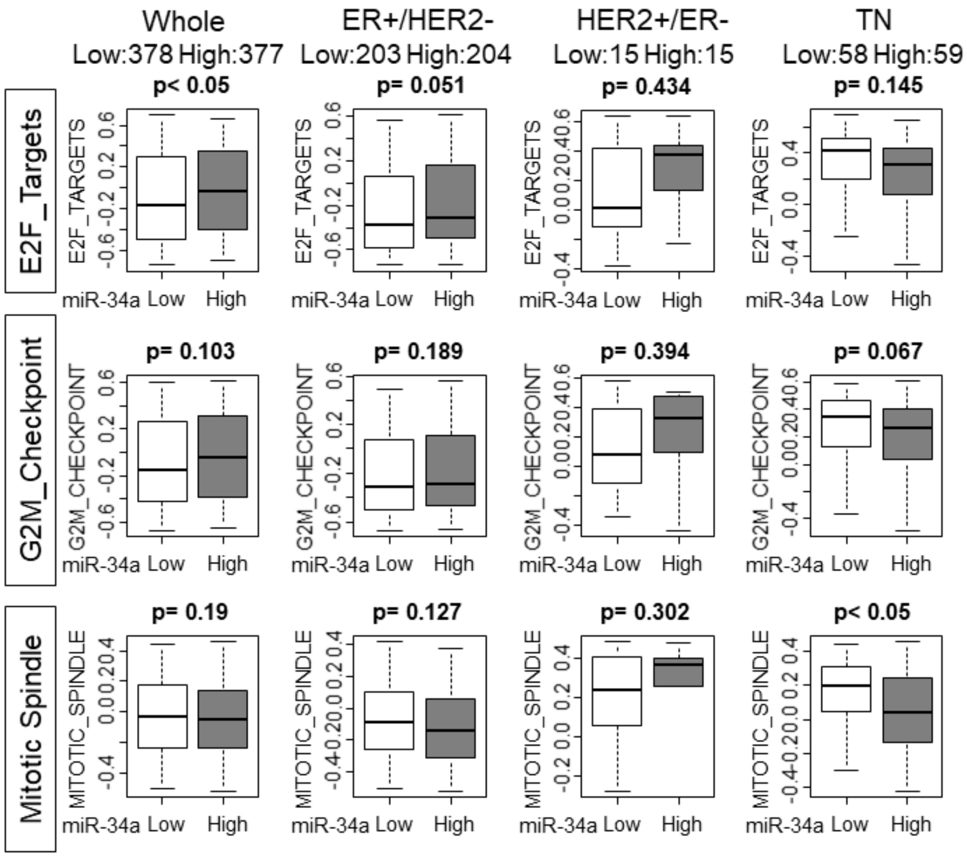

Supplementary Figure S2: The association of High MiR-34a expression with gene sets variation analysis (GSVA) scores of the gene sets related to cell cycle or cell proliferation in whole TCGA cohort and each subtype. Upper row: E2F_Targets, middle row: G2M_Checkpoint, lower row: Mitotic_Spindle Hallmark gene sets. GSVA, Gene Set Variant Analysis.

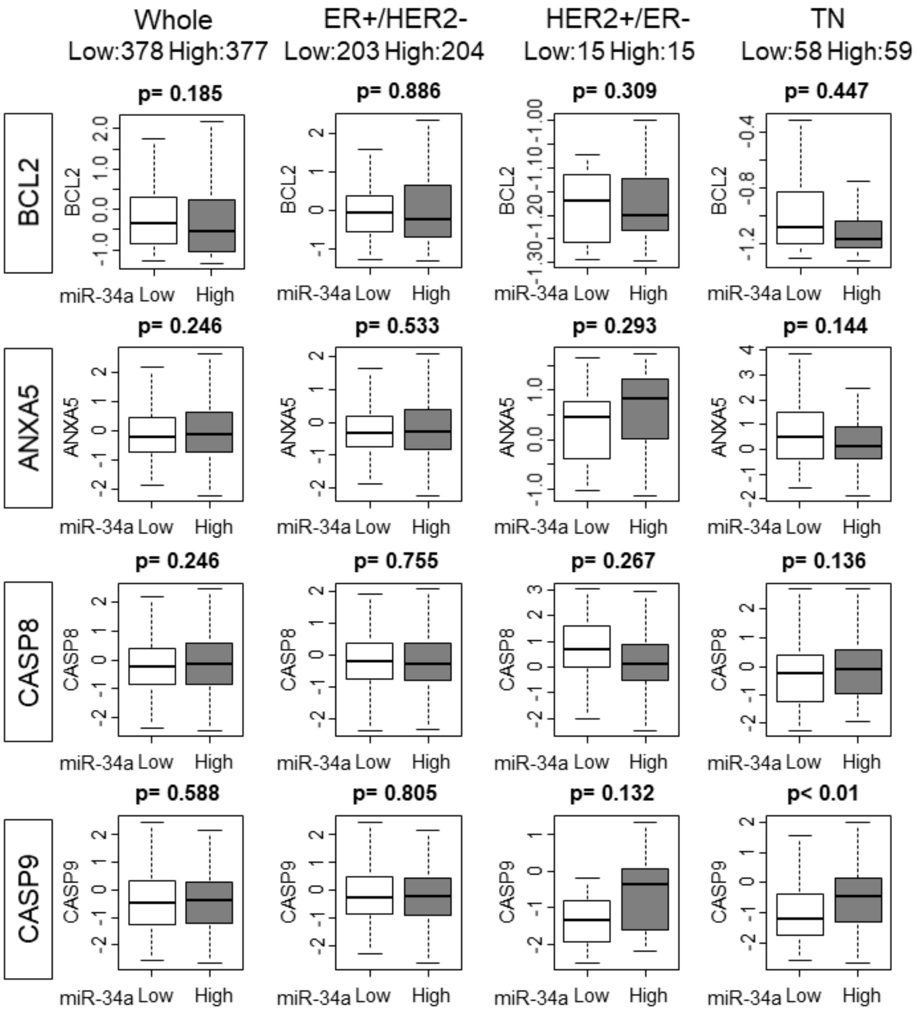

Supplementary Figure S3: The association of High MiR-34a expression with the expression of apoptosis-related genes in whole cohort and ER+/HER2-, HER2+/ER-, and TN subtypes of TCGA cohort.

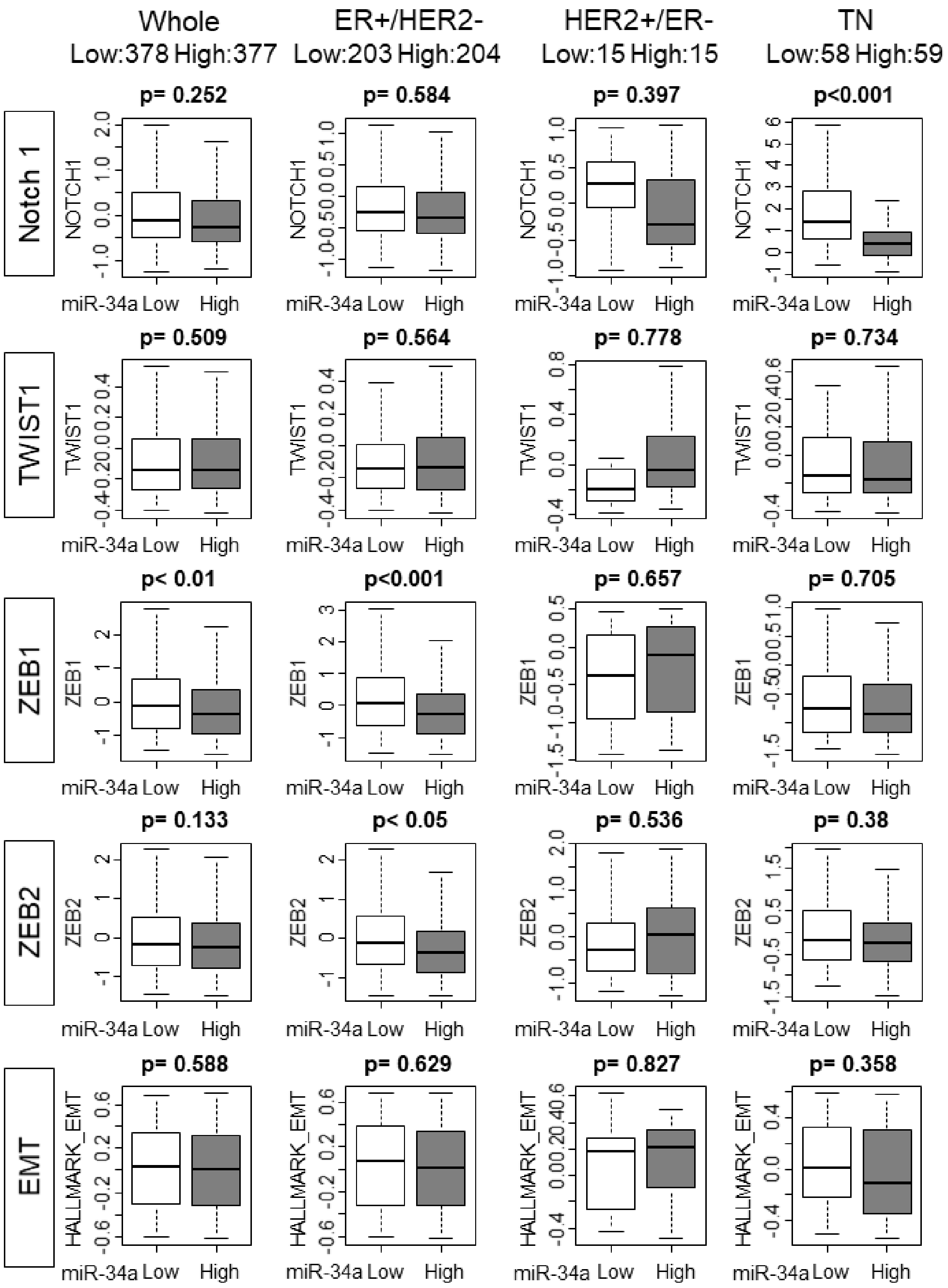

Supplementary Figure S4: The association of High MiR-34a expression with the epithelial–mesenchymal transition (EMT) score. Each row has TCGA whole cohort, and each subtype. Top row: Notch 1 expression by miR-34a expression. Second row: TWIST 1 expression by miR-34a expression. Third row ZEB 1 expression by miR-34a expression. Fourth row: ZEB 2 expression by miR-34a. Bottom row: GSVA Hallmark EMT score by miR-34a expression. EMT, epithelial–mesenchymal transition.

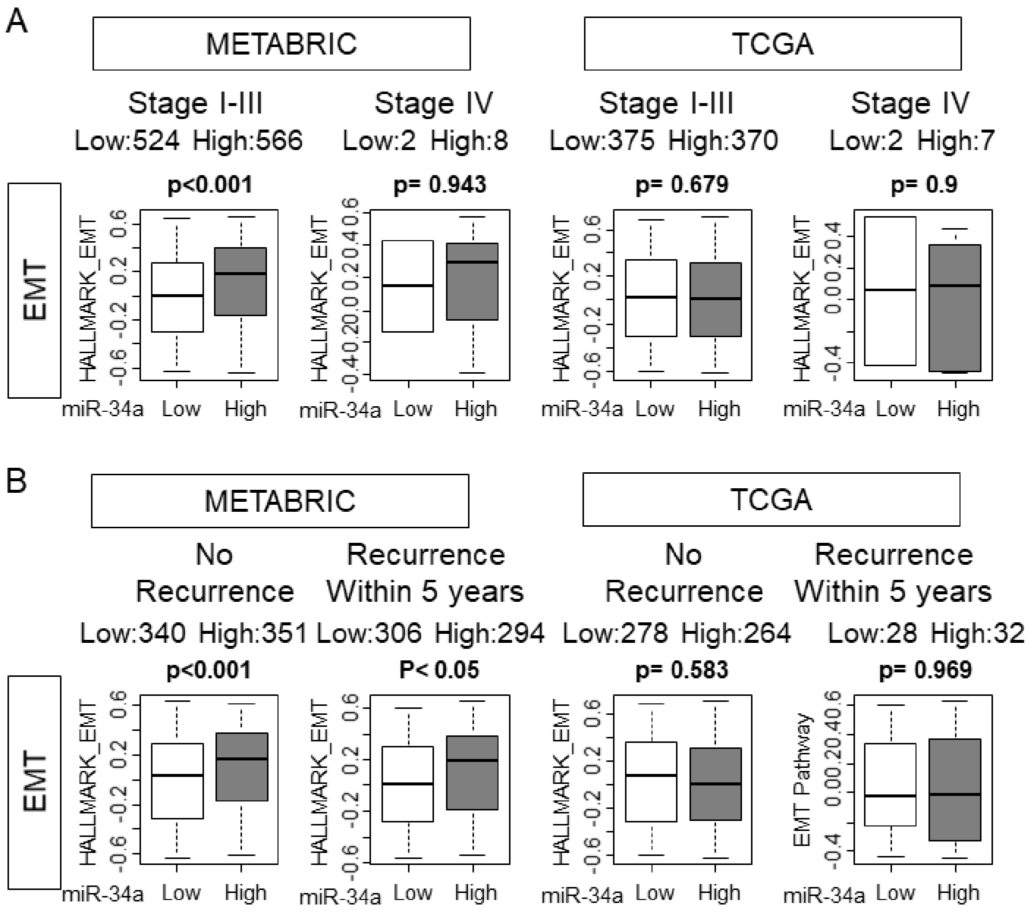

Supplementary Figure S5: High MiR-34a expression tumors were associated with higher epithelial–mesenchymal transition (EMT) score with the patients with Stage I-III, no recurrence over 5 years, recurrence within 5 years. (A) EMT expression by miR-34a expression
